# Detection of Internal Metal Loss in Steel Pipes and Storage Tanks via Magnetic-Based Fiber Optic Sensor

**DOI:** 10.3390/s18030815

**Published:** 2018-03-08

**Authors:** Safieh Almahmoud, Oleg Shiryayev, Nader Vahdati, Paul Rostron

**Affiliations:** 1 Department of Mechanical Engineering, Khalifa University of Science and Technology, P.O. Box 2533, Abu Dhabi, UAE; sajalmahmoud@pi.ac.ae (S.A.); nvahdati@pi.ac.ae (N.V.); 2 Chemistry Department, Khalifa University of Science and Technology, P.O. Box 2533, Abu Dhabi, UAE; prostron@pi.ac.ae

**Keywords:** corrosion sensor, fiber optics, magnets, internal corrosion, pipelines, fiber bragg gratings

## Abstract

A monitoring solution was developed for detection of material loss in metals such as carbon steel using the force generated by permanent magnets in addition to the optical strain sensing technology. The working principle of the sensing system is related to the change in thickness of a steel plate, which typically occurs due to corrosion. As thickness decreases, the magnetostatic force between the magnet and the steel structure also decreases. This, in turn, affects the strain measured using the optical fiber. The sensor prototype was designed and built after verifying its sensitivity using a numerical model. The prototype was tested on steel plates of different thicknesses to establish the relationship between the metal thickness and measured strain. The results of experiments and numerical models demonstrate a strong relationship between the metal thickness and the measured strain values.

## 1. Introduction

Corrosion is one of the most widespread and costly phenomena that affects various sectors of the global economy. Corrosion can be defined as the deterioration of the metallic material as a result of environmental chemical reactions. It is considered a very serious problem as it causes damages to properties and environment, in addition to high costs of mitigation. According to a study conducted by the National Association of Corrosion Engineers (NACE) in 2002, the total estimated annual cost of corrosion in the United States has reached approximately $276 billion [1]. A large portion of overall corrosion costs is attributed to the energy sector, more specifically, the oil and gas industry, which operates an extensive network of pipelines and associated facilities such as storage tanks and processing plants.

In general, corrosion processes may be classified as external and internal. External corrosion refers to deterioration processes that occur on metal surfaces that are exposed to outdoor environments and are largely driven by atmospheric and climatic conditions. Internal corrosion processes occur on the surfaces inside the pipes or storage tanks. Currently, most common corrosion mitigation techniques are based on applying protective coatings on exposed surfaces, and utilization of cathodic protection systems.

External corrosion is usually quantified by monitoring atmospheric corrosivity factors such as relative humidity, temperature, time of wetness, and industrial pollutants (e.g., SO${}_{2}$, NO${}_{x}$) [2,3]. Others are based on weight loss measurements of coupons exposed to the environment. The authors have also recently proposed a passive sensor powered by wireless energy transfer for quantification of corrosivity of atmospheric environment [4,5].

Internal corrosion is much more difficult to detect and quantify. In this work we focus on development of a sensing solution aimed at detection of internal corrosion. First, we describe some conventional technologies that are used to detect and quantify internal corrosion.

Large oil and gas transportation pipelines are usually inspected using special tools (Pipeline Inspection Gages, PIGs) that are launched into the pipe. They are mainly used for cleaning the pipeline by pushing it along the length of the pipe with the pressure-driven flow of the product inside the pipeline from the launching station to the retrieving station. Nowadays, PIGs are capable of providing data about the condition of the pipelines by utilizing several technologies that are able to internally inspect the pipelines for surface defects such as pits, cracks, or corrosion [6].

A technique called Magnetic Flux Leakage (MFL) is used by the PIGs to detect and characterize corrosion in pipes, which effectively represents metal loss. It is based on magnetizing the walls of the pipes, and when the PIG passes through an area with corrosion or other anomalies, the magnetic flux leaks with a greater amount away from the wall. This leak is detected by a sensor, that provides a measure of the thickness of the metal in that area [7].

Other techniques that are used to quantify corrosion and metal loss include ultrasonic technology [6], hydrostatic testing [6], and even optical inspections [8]. The Ultrasonic Technology tool (UT) is another inspection tool used for corrosion detection in pipelines. It provides similar data as the MFL PIG tool. It can directly quantify the thickness of the pipe wall by transmitting an ultrasonic pulse into the wall and processing the reflected signal [6]. However, it is not very commonly used as the MFL tool due to the fact that the pipeline wall must be clean in order for the tool to provide accurate measurements.

Although PIGs are widely used, they are very expensive and cannot provide measurements on demand at any time. Also, since smart PIGs should enter the pipeline from a launching station and go to a retrieving station, the pipeline should be put out of service during the inspection operation. Moreover, major modifications should be done on the pipeline before using these PIGs. Many pipelines are of relatively small diameter and do not have launching and retrieval facilities.

The MFL-based inspection techniques such as the one described by Ref. [7] requires large amounts of electrical power to activate the electromagnets. Again, the pipelines must be piggable and have all relevant facilities in place. The inspection will also put the pipeline temporarily out of operation.

As for the ultrasonic technology, it also has some limitations on its uses. Some pipelines cannot be inspected by the UT tool, such as crude lines with paraffin build up. This is because the inspected pipeline wall should be clean in order for the UT tool to be effective. Also, the UT tool performs well when applied for heavy-wall pipes as opposed to thin-wall pipes. Furthermore, a suitable coupling medium is required between the substrate and the probe when using this technology.

Corrosion probes based on galvanic sensing require power supply, so they are considered as a source of risk for oil and gas pipes. Also, installation of these probes implies performing major modification to the pipeline system and they can provide local measurements only.

Finally, hydrostatic testing requires putting the pipeline out of operation, and using large amounts of water during the test, which might be difficult to obtain in some regions such as the UAE. After the test, the used water should be treated before disposal because it contains toxic petroleum products. This treatment introduces significant additional costs.

The ideal sensing solution must possess the following characteristics in order for it to be widely accepted and implemented by the industry [4,5]:

- The sensor must not require a continuous power supply. In other words, it should be passive. This ensures that it will be safe in volatile environments such these near oil and gas pipelines.
- The sensor must not interfere with existing pipe structure. It shall not require stopping transportation of hydrocarbons through the pipe during installation and operation.
- The proposed sensor should be inexpensive considering that a large quantity of sensors will be necessary to instrument any realistic pipeline.
- Installation and replacements costs must be low.

To conclude, all the above mentioned internal corrosion monitoring techniques do not meet the required constraints and objectives of this research, especially the need for passive sensing over relatively long distances. This research aims to develop a monitoring solution for detection of metal loss for pipelines or storage facilities that are difficult to inspect internally with conventional tools. Typically, unpiggable pipelines are the ones with small nominal diameters, such as flowlines and gathering lines in the oil fields.

The authors believe that the most suitable sensing technology for such application will be based on optical fibers. Optical fiber sensors have several features which give them an advantage over other types of sensors. They are lightweight, compact, small, economically feasible and can be conveniently multiplexed on a single fiber network [9,10,11]. Also, they are immune to electromagnetic interference (EMI) as the sensing point does not contain any electrical currents. Moreover, fiber optic sensors are resistant to corrosive and harsh environment and can be easily embedded into or attached to the structures with a very small modification on the structures [9,10,11].

A special feature of the fiber optics sensor is that it requires no wires to connect to the control system as fibers themselves act as both the sensing element and the signal propagation channel [11]. As a result of all these features, techniques based on the fiber optic sensing technology may be able to achieve above mentioned objectives of this research. There are many different uses of optical fibers as sensors, here we present a short review of optical fibers used for corrosion sensing and monitoring.

Gao et al. [12] developed a fiber optic corrosion sensor (FOCS) to monitor the corrosion rate in reinforced concrete structures. The sensor was made of a fiber Bragg Grating (FBG) and two identical steel rebar elements. When the two elements corrode, their volume is expanded causing strain and resulting in a shift in the FBG peak wavelength. However, the FBG is very sensitive not only to strain, but also to temperature. Thus, an unstrained fiber optic temperature sensor (FOTS) was also built to compensate for the temperature effect. In other words, to remove the contribution in the measured strain caused by temperature variations.

McCague et al. [13,14] developed another sensor for detection of corrosion of reinforcement bars in concrete. Their concept relies on pressure sensing technique based on the polarization characteristics of a polarization-maintaining photonic crystal fibers (PCF). As the steel bar corrodes, corrosion products end up occupying a larger volume and generating internal forces in the concrete, hence increasing the pressure on the fiber.

Al Handawi et al. [15] presented a strain based FBG sensor for nearly real-time corrosion rate monitoring in pre-stressed structures. The proposed sensor detects environmental corrosion and is considered valuable because of its ability to measure corrosion rate in nearly real-time, while being safe, which is usually required by industrial standards applicable in volatile environments.

Jiang et al. [16,17] developed a real-time internal corrosion monitoring technique based on the optical frequency domain reflectometry (OFDR) technology. They used this technology along with signal processors to provide high-resolution measurements of hoop strain in pipes using fiber optics. The technique claims to be capable of measuring strain with millimeter scale resolution and a precision of microstrain. The solution includes the detection of corrosion severity and location for both uniform corrosion and local corrosion in pipelines. The internal pressure and pipe diameter are usually constants, thus the strain measured using the fiber optic can be directly related to the change in wall thickness as a result of internal corrosion. As the wall thickness decreases, the measured hoop strain shall increase. Zou et al. [18] also utilized optical fiber strain and temperature sensors to detect reduction in the wall thickness by measuring hoop strain.

Jaffrezic-Renault and Benounis [19] developed an optical fiber sensor to measure corrosion in the metallic structure of aircraft. The purpose was to re-evaluate the service life and durability of aircraft especially when they exceed their initial design life. The sensor should help detect corrosion in its early stages without the need for disassembly in order to reduce the maintenance cost. The sensor was assembled by first removing the cladding of the fiber optic cable where the core becomes exposed. A metallic material, either aluminum or copper, was then used to cover the core and replace the cladding utilizing a deposition method. The aluminum was deposited by thermal evaporation using a vacuum chamber, while the copper was deposited by an electrochemical method where it is immersed in a saturated metal solution. When corrosion occurs, part of the metal cladding is removed and metallic ions are added into the solution. After conducting the experiment, it was observed that the reflected light intensity changes as the metal corrodes. A similar sensor utilizing carbon steel film has been developed by Ref. [10].

Another sensor for detection of corrosion under coatings and insulation was developed by Deng et al. [20]. Corrosion that occurs under a soft coating causes the coating to deform since corrosion products tend to occupy larger volume, which induces the strain measured by the FBG sensor.

Pacheco and Bruno [21] proposed and built a non-contact sensor which utilizes the FBG technology and the magnetic attraction force to measure external corrosion. The force is induced between a small permanent magnet and a magnetic material (i.e., targeted metal) which is placed few millimeters away from the magnet. As the metal corrodes, the distance between the permanent magnet and the magnetic material increases as a result of the material removal. Thus, the attraction force between the metal and the magnet decreases resulting in a decrease in the strain, which is measured by the optical FBG sensor. Somewhat similar concept has been described in a more recent work by Li et al. [22], where they utilized a magnet and a spring. Again, the working principle of that sensor relied on the change in the distance between the magnet and the steel surface facing the magnet due to external corrosion.

In this research, we will utilize similar physical and sensing principles as in Ref. [21,22], i.e., using the magneto-static force and fiber optic strain sensing technology. The working principle of the proposed sensor is based on the magneto-static force, but is different than the one used by [21,22] as changes in the force will not depend on the distance change between the magnet and the metal surface facing it, but rather on the change in the thickness of the metal structure due to corrosion occurring on the opposite surface. We envision that the sensor proposed in this work will be a first step toward the solution that is able to provide on-demand measurements for monitoring of internal corrosion rates in structures such as unpiggable pipelines.

## 2. Sensor Design and Analysis

Most oil and gas pipes and storage tanks are made of carbon steel, which is a highly ferromagnetic material. Internal corrosion causes a decrease in the pipe wall thickness on internal surface; which, according to the principle shall reduce the force generated between the magnet and the pipe. The magnitude of this magnetic force can be easily related to the strain. Thus, as the wall thickness decreases, the strain measured by the optical fiber shall also decrease.

**Figure 1:**
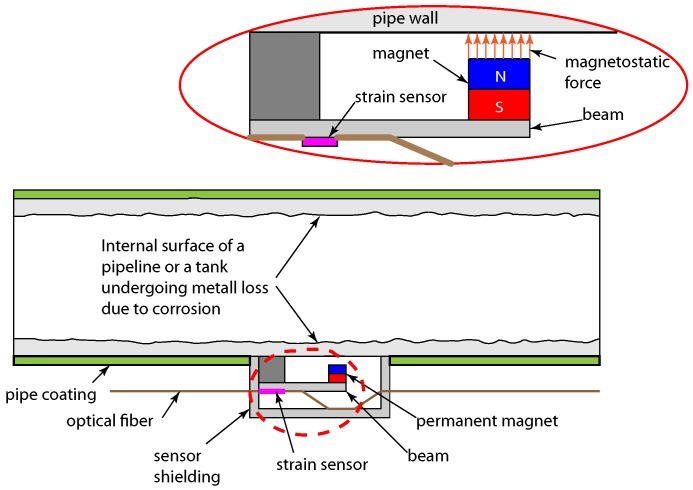
Initial schematic of proposed sensing system.

The main parts of the sensor include an elastic beam made of a non-corrosive material, and a strong rare-earth permanent magnet attached to the beam. An optical strain gage is also attached on the beam’s surface to measure the strain. The metal loss sensor will be designed in a way such that, when attached to the pipe, the magnet becomes very close to the pipe. This will allow for the generation of a magneto-static force between the pipe wall and the beam, which is a force that pulls the beam towards the wall of the steel pipe. An initial schematic of the proposed sensing system is shown in Figure 1.

After the outside surface of the pipe is cleaned, the sensor should be attached at the bottom of the pipe since it is the area where most of the internal corrosion usually occurs. Pipe cleaning includes removing the pipe coating on the area where the sensor is to be mounted. This step is important if the coating was thick (e.g., more than 0.5 mm), so that the sensor’s magnet is kept only few millimeters away from the pipe wall. After cleaning, the sensor shall be mounted on the exposed metal using a suitable adhesive.

As the pipe or the storage tank wall starts to corrode, some material on the internal surface is removed, so the thickness of the wall will decrease. Therefore, the magneto-static force between the sensor and the pipe will be smaller. The change in the force magnitude, which can be calculated using the Maxwell stress tensor, can be easily related to the change in the strain that is measured by the optical strain sensor attached to the beam. It is important to note that the beam is not pre-stressed until the magnet is placed near the pipe wall.

To ensure accurate measurements, the sensor components shall be surrounded by a housing or shielding, which is made of a non-corrosive material, e.g., heavy duty UV-stable plastic. This housing will enclose the entire sensor except on the side and location where the magnet is supposed to face the pipe wall, and allow the optical fiber to pass through it. The enclosure shall be sealed at the interface with the pipe wall using a suitable adhesive sealant. The enclosure is intended to eliminate the external corrosion factors, and therefore allow to detect the internal corrosion accurately. This sensor should be able to detect internal corrosion over the entire pipeline length. One long fiber with multiple sensors arranged in series can be deployed over the entire length of the pipeline.

### 2.1. Magnet Selection

It was decided to use Samarium-Cobalt (SmCo) rare earth magnets in the sensor development as they possess much wider working temperature range compared to Neodymium magnets (up to 250 °C compared to 80 °C). In order for this sensor concept to become viable for the actual application, the magnet dimensions need to be much larger than the metal wall thickness. Considering a typical wall thickness of about 6–7 mm, the magnet characteristic dimensions should be at least 20 mm. The larger magnets are expected to provide earlier detection of thickness change, thus being able to provide a warning at an early stage. For this sensor prototype design, a large cylindrical Samarium-Cobalt Sm2Co17 (grade 30) rare-earth permanent magnet of 1” diameter and 1” height will be used [23,24].

### 2.2. Preliminary Magnetostatic Force Analysis

To assess the capabilities of this magnet and estimate values of the force expected to act between the magnet and steel plates, a finite element (FE) model was developed and run for different cases of plate thicknesses and separation distances. COMSOL Multiphysics [25] software with AC/DC electromagnetic analysis module was used for this purpose.

**Figure 2:**
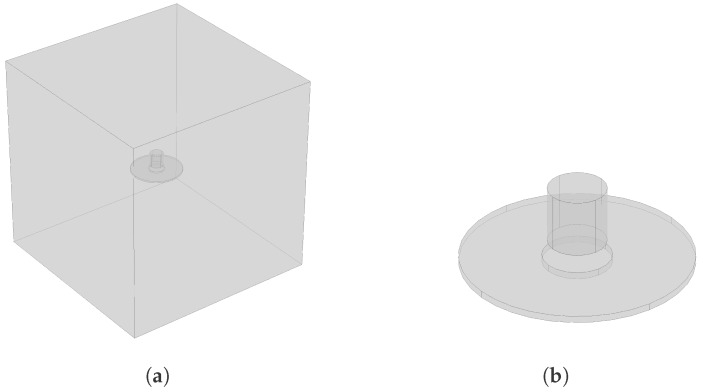
(**a**) Geometry of the finite element (FE) model with the 25.4 × 25.4 mm cylindrical magnet; (**b**) Close-up view of the geometry with the cylindrical magnet in the FE model.

The model geometry consists of the 1”×1” cylindrical Samarium-Cobalt magnet and a disc-shaped plate made of carbon steel. These parts are surrounded by a cuboid air box as shown in Figure 2a. A close-up view of the magnet and disc geometry is shown in Figure 2b. The steel disc was partitioned into 2 concentric parts, so that a finer mesh is assigned to the central part near the magnet. The central part of the disc is cylindrical and has a radius of 15 mm. The following parameters were assigned in the model:

***Steel disc***: Radius (*R*): 50 mm; Thickness (*T*): Varying from 1 to 9 mm.
***Magnet***: Diameter (*D*): 25.4 mm; Height (*H*): 25.4 mm.
***Air Box***: Depth = Width = Height = 0.4 m.
***Separation distance between magnet and disc*** (*d*): Varying from 0.1 to 7 mm.

The mesh size in this model was set to 0.3 mm for the magnet area and the center part of the disc, while the default “extra fine” mesh size option was assigned for the rest of the geometry. The steel disc material was assigned with a relative permeability $\mu_{r}$ value of 100. The remanent flux density ($B_{r}$) value for this magnet is 1.05 Tesla, which was used in the model. The model was parametrized to enable quick change of the separation distance (*d*) between the magnet and the disc, and the disc thickness (*T*).

**Figure 3:**
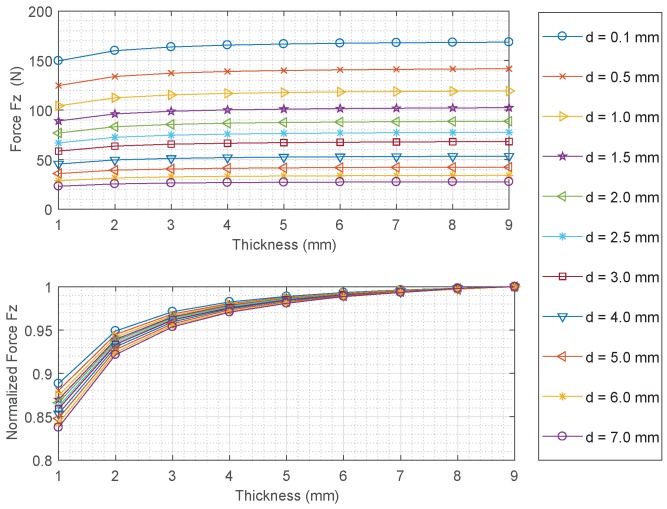
Magneto-static force predicted by the FE model with the large cylindrical magnet.

Simulation results are presented in Figure 3. The top plot represents the magnitude of force and the bottom plot represents the normalized force magnitude for each value of separation distance *d*. From presented results one can conclude that the magnitude of force increases as the separation distance is decreased (the magnet gets closer to the steel disc). The force magnitude decreases as the thickness of the disc decreases. This is an important finding supporting the main operating principle of the proposed sensor.

### 2.3. Beam Configuration

In order to devise the sensor design with high sensitivity to the change metal thickness, multiple considerations need to be taken into account. First, the selected beam configuration shall provide the highest strain value and the highest sensitivity to force changes. This is particularly important to ensure that the sensor is capable of detecting very small changes in strain values. The optimum location on the beam for application of the force due to the magnet, which gives the highest strain value shall be identified. It is also important to determine the best location for attaching the optical fiber strain sensor on the beam.

**Figure 4:**
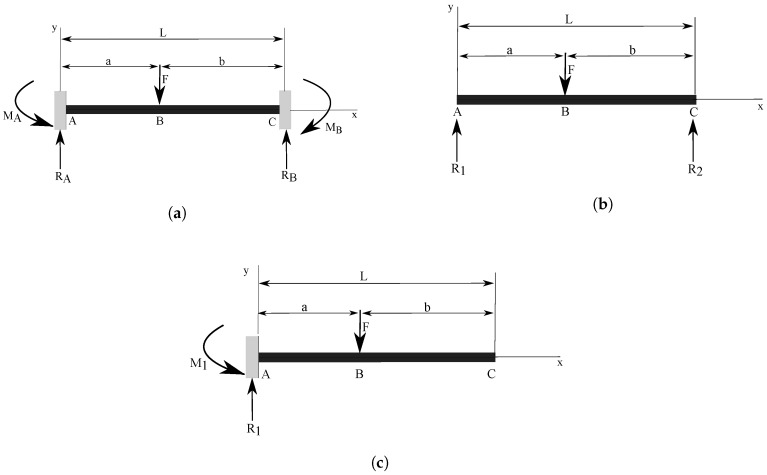
Beam configurations considered for the sensor design. (**a**) Fixed-Fixed beam structure with a concentrated load; (**b**) Simply supported beam structure with a concentrated load; (**c**) Cantilever beam structure with a concentrated load.

We considered the relationships for the normal strain and deflection on the top surface of a straight beam for three different boundary conditions: fixed-fixed, simply supported, and a cantilever shown in Figure 4a–c. These relationships can be derived using the principle of superposition and the Euler-Bernoulli beam theory, which are covered in majority of books on mechanics of materials, for example Refs. [26,27,28].

The normal bending stress $\sigma_{x}$ produced in a straight beam by a bending moment *M* is given by the following known formulation:

(1) $$\sigma_{x}=\frac{My}{I}$$

where *I* is the moment of inertia (or second moment of area) of a beam’s cross section, and *y* is the distance from the neutral axis to the beam surface. Equation (1) is known as the elastic flexure formula [26]. The normal strain ε can be easily determined by Hooke’s Law:

(2) $$\varepsilon =\frac{\sigma }{E}$$

where *E* is the elastic modulus of the beam’s material. We proceed to analyze the stress and strain in three different beam configurations as shown in Figure 4.

For the fixed-fixed beam configuration, the normal stress as a function of axial coordinate is expressed as:

(3) $$\sigma_{AB}=\frac{Mc}{I}=\frac{M}{S}=\frac{\frac{Fb^{2}}{L^{3}}\left[x\left(3a+b\right)-aL\right]}{S}=\frac{Fb^{2}}{L^{3}S}\left[x\left(3a+b\right)-aL\right],\;\;\;0⩽x⩽a$$

(4) $$\sigma_{BC}=\frac{Mc}{I}=\frac{M}{S}=\frac{M{\left(x\right)}_{AB}-F\left(x-a\right)}{S}=\frac{\frac{Fb^{2}}{L^{3}}\left[x\left(3a+b\right)-aL\right]-F\left(x-a\right)}{S},\;\;\;a⩽x⩽L$$

For the simply supported beam configuration, the normal stress as a function of axial coordinate is expressed as:

(5) $$\sigma_{AB}=\frac{Mc}{I}=\frac{M}{S}=\frac{Fbx}{SL},\;\;\;0⩽x⩽a$$

(6) $$\sigma_{BC}=\frac{Mc}{I}=\frac{M}{S}=\frac{Fa}{SL}\left(L-x\right),\;\;\;a⩽x⩽L$$

For the straight cantilever beam configuration, the normal stress as a function of axial coordinate is expressed as:

(7) $$\sigma_{AB}=\frac{Mc}{I}=\frac{M}{S}=\frac{F(x-a)}{S},\;\;\;0⩽x⩽a$$

(8) $$\sigma_{BC}=\frac{Mc}{I}=\frac{M}{S}=\frac{-Fa+Fx-F(x-a)}{S}=\frac{0}{S}=0,\;\;\;a⩽x⩽L$$

where *F* is the applied force, *c* is the distance from the neutral axis to the outermost fiber, and *S* is the section modulus of the cross-section of the beam.

In order to determine which straight beam configuration provides the maximum strain value, we consider an example case. We assume a straight beam of length L=0.2 m, that has a rectangular cross section with the following parameters: width b=0.005 m, and thickness/height h=0.008 m. We consider a beam made of ABS plastic with elastic modulus E=3 GPa. A point force F=30 N that represents a load due to the magnet interacting with a steel plate is applied to the beam. For each configuration, we vary the position of the applied load a∈[0,L] and plot the strain values at the different locations x∈[0,L] along the beam with the varying values of *a*.

**Figure 5:**
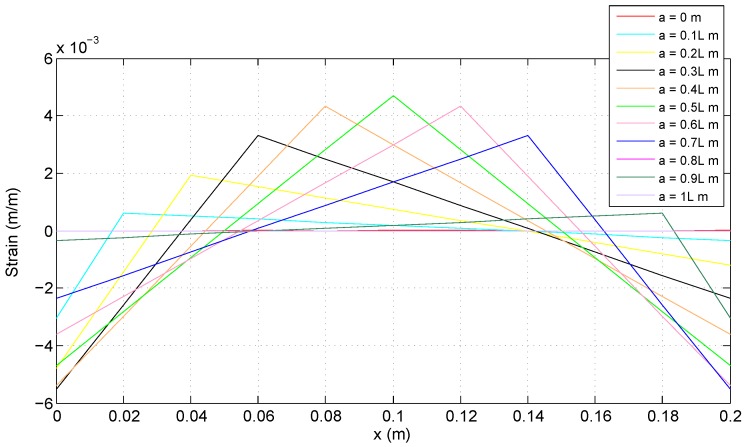
Strain values for the fixed-fixed beam structure.

Figure 5 illustrates strain distribution and magnitude for several different positions of the applied force the case of the fixed-fixed beam. The largest strain magnitude occurs at the fixed ends when the applied load is located between x=0.3L and x=0.4L, or symmetrically between x=0.6L and x=0.7L.

For the simply supported beam configuration the largest magnitude strain occurs when the point load is applied in the center of the beam span. For the straight cantilever beam with a point load at the free end the largest strain occurs at the fixed end of the beam.

Beam configuration which provides the maximum magnitude of strain will also be the most sensitive to force changes as the thickness of the steel plate decreases. To illustrate this, we take the same example where a 30 N force is applied, and compare the maximum strain value to the same case, but when the applied force is reduced by 2% (F=29.4 N). We calculate the differences in corresponding strain values between the two cases. The results are shown in Table 1. It can be clearly seen that the largest magnitude strain difference occurs in the case of the straight cantilever beam. Thus, the straight cantilever beam is considered as the most sensitive to force changes out of all the three configurations discussed in this paper. Therefore, we chose to use the straight cantilever setup in the sensor prototype.

### 2.4. Sensor Prototype Analysis

After selection of the beam configuration, its dimensions have to be determined. Multiple constraints are considered here. The maximum strain on the beam must be within the range of ±5000 με, which is the safe operating range of the strain gauge used [29]. Also, the beam deflection (in y direction) must be less than 5% of the beam length, to maintain the accuracy of the analytical solution. The material chosen for the beam is the Aluminum alloy (6063-T6), due to the fact that it is a non-magnetic material readily available in the lab and has a relatively high yield strength value for an aluminum alloy. The mechanical properties of this material are: yield strength $\sigma_{y}$ = 215 MPa; Young’s modulus E=69 GPa; shear modulus G=25.8 GPa.

**Figure 6:**
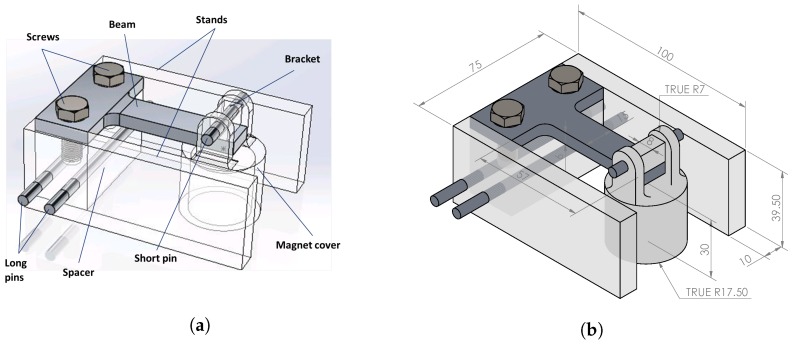
Sensor prototype details. (**a**) Sensor prototype parts; (**b**) Main dimensions of the prototype (mm).

**Figure 7:**
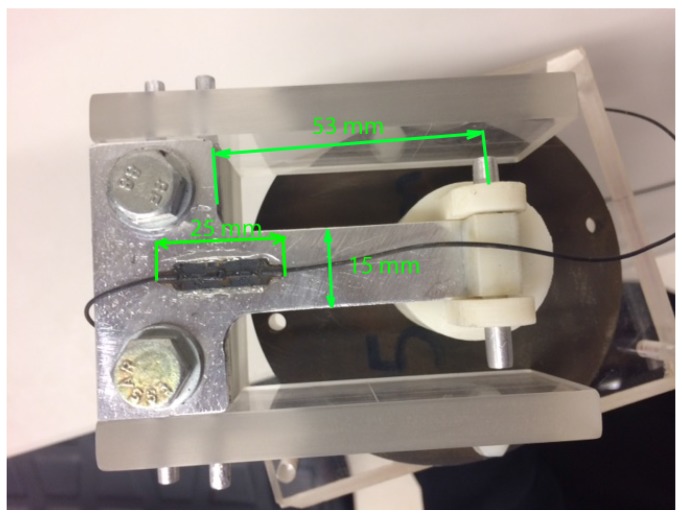
Optical strain sensor mounted on the beam of the sensor prototype.

The final design of the sensor prototype is shown in Figure 6. Using the analysis tools described in the previous section we chose dimensions for the aluminum beam to be: effective cantilever length L=0.053 m, width b=0.015 m, and thickness h=0.006 m, see Figure 7. The prototype consists of the above mentioned beam clamped to a spacer by two screws. The spacer is attached to the mounting stands by two long pins. The stands serve the function of supporting the sensor on the steel plate. A bracket made of ABS plastic is attached to the free end of the beam. The bracket holds a pin, which supports the cylindrical plastic cover, which, in turn, houses the magnet.

We perform stress, strain and deflection analysis, to ensure that chosen beam dimensions result in stress, strain and deflection values within acceptable limits. To eliminate the chance of the magnet and the steel plate getting in contact, the sensor is designed in a way that the minimum possible stand-off distance between the magnet and the steel plate is 1 mm.

A force value of 120 N is considered in this analysis, which is the maximum force magnitude at 1 mm distance (corresponds to the case with 9 mm steel plate thickness). This force value results in the maximum strain and deflection possible. At larger distances or with smaller plate thicknesses, the value of force will be lower, thus the strain and deflection will definitely be smaller.

Using the strain energy theory and Castigliano’s theorem the total deflection of the free end of the beam in the direction of the load is calculated by combining the effects caused by the bending moment $\delta_{F_{\left(M\right)}}$ as well as the shear loading $\delta_{F_{\left(S\right)}}$ [27,28], as follows:

(9) $$\delta_{F}=\delta_{F_{\left(M\right)}}+\delta_{F_{\left(S\right)}}=\frac{\partial U_{\left(M\right)}}{\partial F}+\frac{\partial U_{\left(S\right)}}{\partial F}=\frac{FL^{3}}{3EI}+\frac{kFL}{GA}$$

where $U_{\left(M\right)}$ and $U_{\left(S\right)}$ are the strain energies due to bending and shear, and k=6/5 is the the form factor for shear of the rectangular cross-section [27,28]. Results of analysis are shown in Table 2. The maximum normal strain is located at the fixed end of the beam, and it was calculated using Equations (2) and (7). The result was found to be $1.024\times 10^{-3}$. However, the actual beam geometry has filleted edges with a 5 mm radius, therefore, a stress concentration factor must be considered in the stress calculation. According to Ref. [30], the stress concentration factor for this geometry is approximately 1.4. This factor was multiplied by the previously obtained strain value, to get a maximum strain value of $1.434\times 10^{-3}$.

The maximum deflection occurs at the free end of the cantilever beam and it was calculated using Equation (9) for thick cantilever beams. Furthermore, the maximum stress on the sensor which is located at the fixed end of the beam was calculated as 98.93 MPa, using Equation (7) and the stress concentration factor of 1.4. This stress value is below the maximum allowable stress value, which was obtained by dividing the yield strength of the aluminum alloy ($\sigma_{y}=215$ MPa) by the safety factor ($f_{s}=2$) to obtain $\sigma_{a}=107.5$ MPa. Calculated stress, strain, and deflection values are well within the allowable ranges. It must be noted that in sensor designs for high temperature applications, one must take into account plastic creep that occurs in materials subjected to stress over long period of time. This effect could be reduced if one chooses materials with high melting temperatures and high strength.

## 3. Experimental Results

To test the feasibility of the proposed sensor, an experiment was designed with an optical fiber sensing gauge, steel specimens of different thicknesses, and a data acquisition instrument. Results were recorded at regular intervals to collect multiple sample readings that were analyzed further. Experimental results are then compared to simulation results.

**Figure 8:**
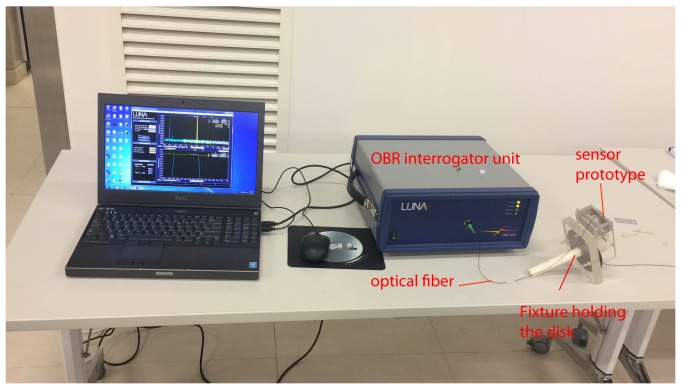
Experimental setup.

The photo of experimental setup is shown in Figure 8. It includes an optical strain gauge that is mounted on the sensor prototype, particularly on the fixed end of the aluminum beam, as shown in Figure 7. The optical strain sensor was attached to the beam using 3M two-part quick set epoxy. The optical strain sensor is located approximately 0.5 m away from the terminal connected into the interrogator.

**Figure 9:**
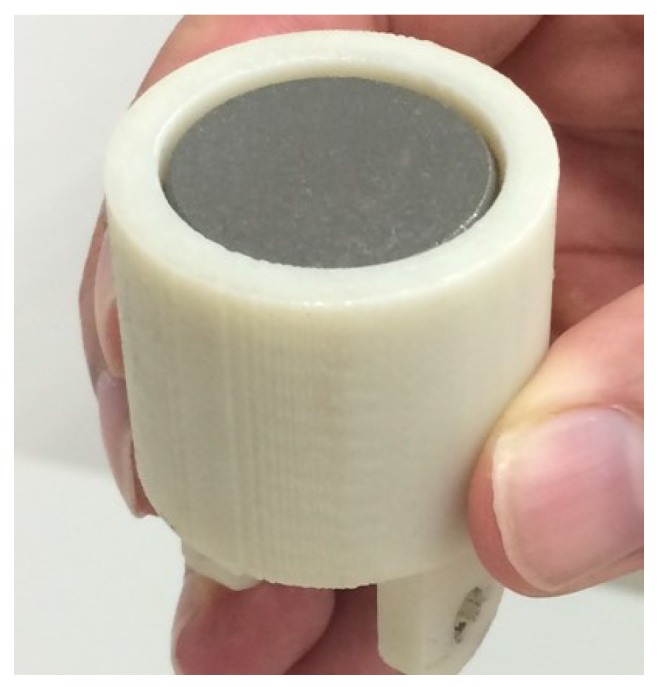
1 mm gap left in magnet cover.

The cylindrical SmCo magnet (1” × 1”) was attached inside the magnet cover using epoxy. After gluing the magnet in the cover, approximately a 1 mm gap in the cover is left, as shown in Figure 9. The importance of this gap is to ensure a minimum separation distance of 1 mm between the magnet and steel plate when the sensor is attached to it, so that they do not get in contact during mounting and removal of the sensor.

The optical fiber with the strain sensor was connected to an optical backscatter reflectometer OBR 4600 [31], which is a reflectometry device with backscatter-level sensitivity for interrogating optical components or systems. The OBR uses swept-wavelength coherent interferometry to measure minute reflections in an optical system as a function of length or position along the fiber. Small random changes in the refractive index along the fiber can be thought of as weak FBGs, and their reflected spectra are sensitive to changes in strain and temperature as a function of position along the fiber. The OBR device performs distributed measurements and in this experiment it is used to measure strain along the optical fiber. The unit is capable of ±1με resolution in distributed measurement mode. The unit was calibrated using a specially supplied reference optical fiber with a gold reflector prior to performing all experiments.

Instead of using an OBR to perform distributed strain measurements, one may also use generic FBG strain gauges and interrogators, which are typically much cheaper. The optical strain gage has a fixture inside which the protective plastic fiber coating is removed exposing the cladding, so the adhesive epoxy comes directly into contact with fiber’s cladding, improving sensitivity to strain that is induced on the structure. In case one wishes to use the OBR in distributed sensing mode, one can also use an unaltered optical communication fiber.

**Figure 10:**
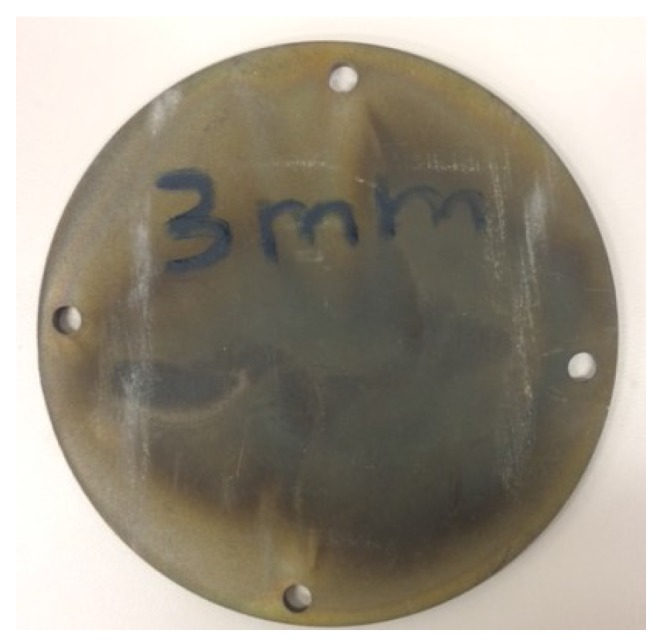
Steel disc sample of 3 mm thickness, 100 mm diameter.

The experimental setup also included a set of carbon steel discs, 100 mm in diameter with nominal thicknesses of 1 mm, 2 mm, 3 mm, 4 mm, and 5 mm. All discs were manufactured by cutting them with wire EDM machine from the one solid round plain carbon steel rod. A sample of the disc is shown in Figure 10. After manufacturing and between experiments all discs were stored in vapor corrosion inhibitor plastic bags.

**Figure 11:**
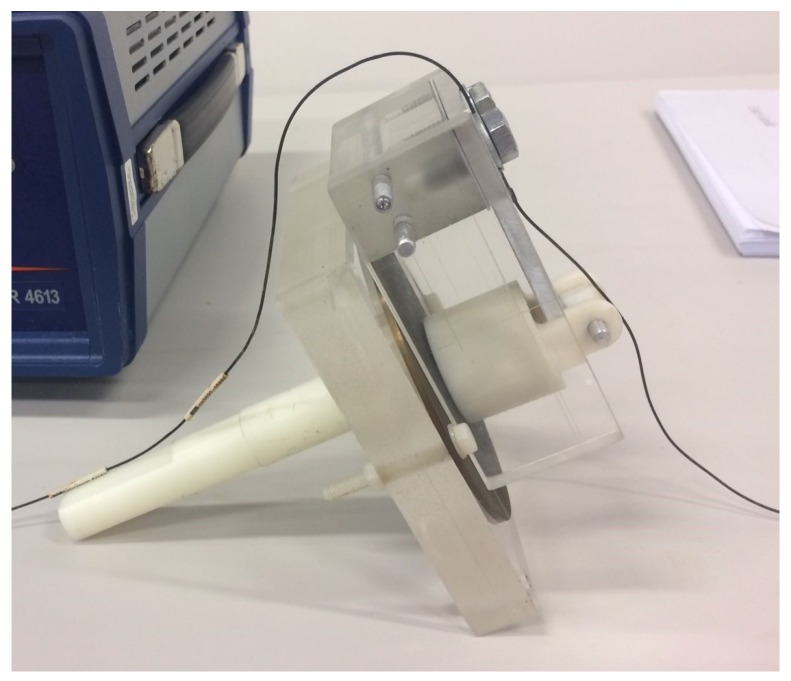
Fixture comprised of an acrylic plate and a nylon rod holds the steel disc with a sensor prototype.

The steel disc is attached to a fixture comprised of an acrylic plate by using nylon screws and nuts, while a nylon rod is glued into a blind hole at the bottom of the acrylic plate. The purpose of this fixture is to hold the steel disc in place while performing the experiment and facilitate installation and removal of the sensor from the steel plates. Fixture materials were chosen to ensure that the only magnetic interaction that occurs during the experiment is that between the magnet and steel plates. The setup is shown in Figure 11.

Prior to performing all experiments, nine readings were recorded and averaged to quantify the amount of noise that can be present in the measurements. The variance of strain measured at the 4 sensing points corresponding to the location of the optical strain gauge is approximately 0.48 με.

**Figure 12:**
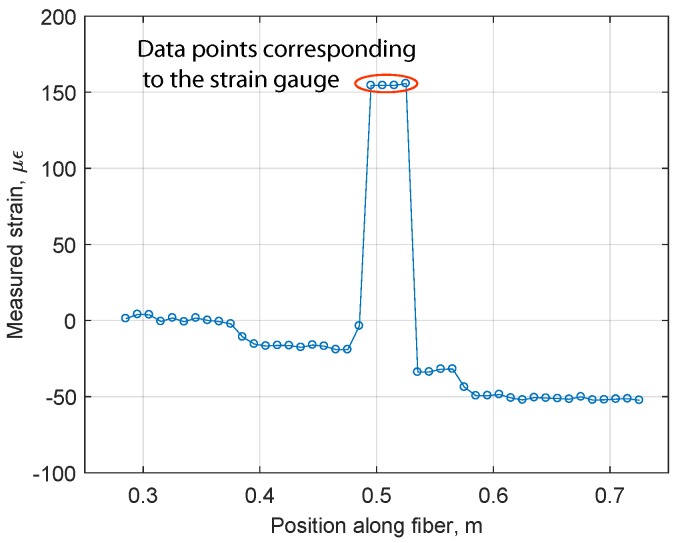
Sample of the measured data, plate of 1 mm thickness.

For each steel disc with a thickness ranging from 1 mm to 6 mm, strain measurements were performed at a regular interval of 30 min during a 4-h period. At each time point six readings were recorded. A sample of the measured data is shown in Figure 12. It can be clearly seen that the peak strain values are located at the position where the strain gauge is located on the optical fiber. This position constitutes 4 adjacent sensing points along the fiber: 0.495 m, 0.505 m, 0.515 m, 0.525 m.

**Figure 13:**
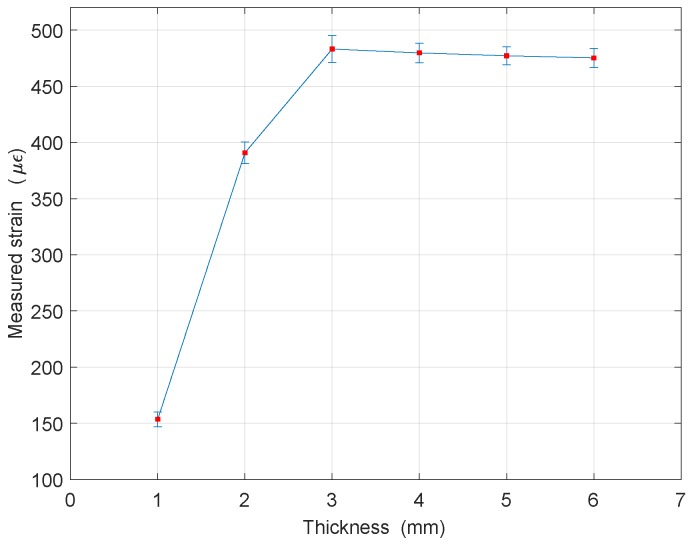
Average strain value acquired for each steel plate thickness.

The collected data for each steel disc thickness were analyzed. Spatial averaging was performed over the 4 sensing points corresponding to the strain gage location to constitute one sample data. Then, the six sample data readings collected at each time point were averaged to obtain one strain value at each time point. The nine average values obtained for the different time points (every 30 min over 4 h) were averaged again to obtain the strain value for each thickness. Figure 13 represents the average strain values acquired for each thickness of steel discs. Errorbars indicate the standard deviations calculated from the measurements.

From Figure 13, it can be clearly seen that the reduction in the steel disc thickness causes a significant decrease in the measured strain value when the thickness becomes smaller than 3 mm. This implies that threshold thickness value is approximately 3 mm. Thus, with this sensor prototype, thickness reduction could be detected once it reduces below 3 mm.

It can also be observed that the measured strain values are not exactly the same, rather they are slightly fluctuating. The major possible reason for this might be due to the variations in the temperature of the surrounding environment. The experiment was performed in a lab with controlled temperature set at 23 ${}^{\circ }$C. Fluctuations of ambient temperature in the lab were observed to be within 1.5 ${}^{\circ }$C. Considering the coefficient of thermal expansion for 6063-T6 aluminum is approximately 20 με/1 ${}^{\circ }$C in this temperature range, and the optical strain gauge data provided by its manufacturer [32], it is possible to calculate the contribution of thermally induced strain in the optical strain sensor. For a temperature variation of 1.5 ${}^{\circ }$C, the resulting strain variation turns out be 40.55 με. From Figure 13 one can observe that standard deviations observed at each thickness value are smaller than 40 με.

Hence, the observed fluctuations in strain readings can be attributed to fluctuations in ambient temperature. Since the strain gauge is sensitive to both strain and temperature, another strain gauge shall be used to compensate for the effect of changing ambient temperature on the strain measured on the beam. In this experiment, due to the unavailability of another optical strain gauge, we were not able to compensate for the temperature effect. The temperature sensor in the lab showed a variation in the environmental temperature of no more than 1.5 ${}^{\circ }$C during all experiments. This indicates that the thermal effect on the strain sensor was relatively small, thus the results can be trusted.

Temperature fluctuations will affect strain readings because optical strain gages are sensitive to both strain and temperature. A common way of accounting for temperature changes is to run a parallel fiber with an unstrained sensor, which will provide the measurement of thermally induced strain that can then be subtracted.

**Figure 14:**
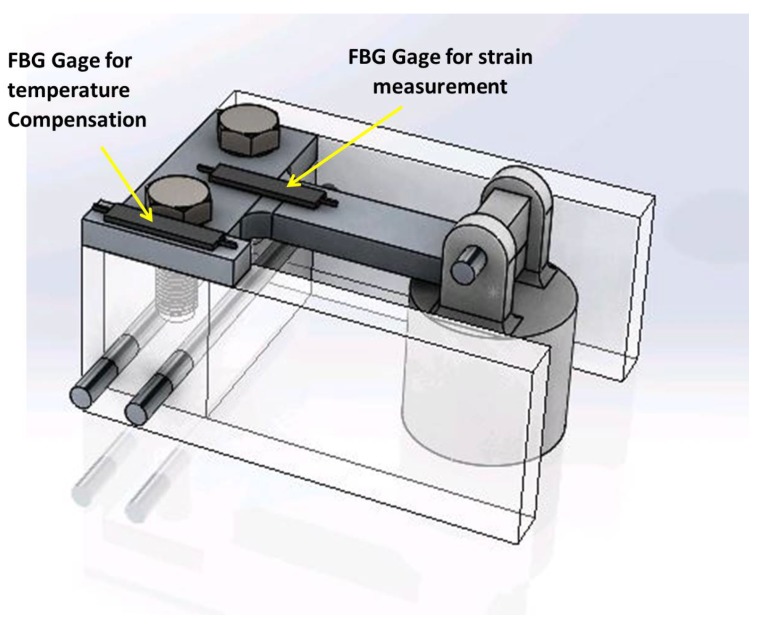
Sensor prototype with proposed temperature compensation.

Strain measurements could have been done using FBG strain sensors that require less sophisticated interrogation equipment. In the case of the sensor proposed in this paper, one can compensate for temperature effects by having a parallel optical fiber, which will have an FBG gauge attached to the unstrained block of aluminum mounted on the same sensor structure, as shown in Figure 14. Alternatively, instead of running a parallel fiber, one may employ multiplexing techniques for FBGs, which allow interrogating large numbers of sensors located on a bus network [33].

## 4. Conclusions

This paper presents a first step in the development of an internal corrosion monitoring technique based on the magneto-static force and the optical fiber sensing technology. The utilized working principle relates the change in metal thickness to the change in magneto-static force, which is related to the measured strain. Numerical simulations and experimentation were conducted to verify the working principle, from which the following conclusions can be drawn:

- Significant decreases in the magneto-static force are observed once the metal plate thickness reduces to a threshold value. Beyond this value, the change in force becomes very large. For steel thickness values larger than the threshold, force values saturate.
- Multiple factors play a role in specifying the threshold thickness value and the saturation force, including: magnet size, material permeability, and separation distance. In case if it is desirable to obtain a direct measurement of wall thickness, one must calibrate the device using exactly the same material as that of the structure in question. Even relatively small changes in magnetic properties of the material due to, for example slightly different iron content, or heat treatment of steel, may cause significant changes in the sensitivity of the device and its measurement range [34].
- Increasing the magnet size results in an increase in the threshold thickness value as well as the saturation force. Therefore, using a larger magnet provides a relatively higher threshold thickness, which means detecting reduction in metal thickness at an earlier stage. Also, based on all simulations and experiments done so far, it has been concluded that in order for the sensor to become viable for actual pipeline application, the magnet dimensions need to be much larger than the pipe wall thickness.
- The magnitude of magneto-static force is highly dependent on the separation distance between the magnet and the steel. Decreasing the separation distance leads to a significant increase in the saturation force magnitude, especially at very small separation distances. On the other hand, results of numerical simulations suggest that the relative reduction in force (threshold thickness) tends to be slightly larger for larger separation distance values; however, this needs to be verified through experimentation in future research work.

## Figures and Tables

**Table 1:** Strain sensitivity in different beam configurations.

Configuration Type	Strain Magnitude for F=30 N	Strain Magnitude for F=29.4 N	Strain Magnitude Difference
Fixed-Fixed	5513 με	5402 με	111 με
Simply Supported	9375 με	9187 με	188 με
Straight Cantilever	37,500 με	36,750 με	750 με

**Table 2:** Results of the analysis of sensor prototype design.

	Analytical Solution	Simulation Result	Constraint
Normal strain	1268 με	1434 με	±5000 με
Deformation	−0.32 mm	−0.46 mm	10% of beam length
Normal stress	98.93 MPa	92.61 MPa	$\sigma_{a}=107.5$ MPa

## Abbreviations

The following abbreviations are used in this manuscript:

FBG	fiber Bragg grating
NACE	National Association of Corrosion Engineers
PIGs	Pipeline inspection gauges
MFL	Magnetic flux leakage
PCF	Photonic crystal fibers
UT	Ultrasonic technology
CCTV	Closed-circuit television
FOCS	Fiber optic corrosion sensor
FOTS	Fiber optic temperature sensor
DOAJ	Directory of open access journals
EMI	Electromagnetic interference
UAE	United Arab Emirates
OFDR	Optical frequency domain reflectometry
FE	Finite element
ABS	Acrylonitrile butadiene styrene
OBR	Optical backscatter reflectometer
EDM	Electrical discharge machining
